# Alterations of White Matter Network Properties in Patients With Functional Constipation

**DOI:** 10.3389/fneur.2021.627130

**Published:** 2021-03-24

**Authors:** Ma Peihong, Yin Tao, He Zhaoxuan, Yang Sha, Chen Li, Xie Kunnan, Chen Jingwen, Hou Likai, Teng Yuke, Guo Yuyi, Wang Fumin, Tian Zilei, Sun Ruirui, Zeng Fang

**Affiliations:** ^1^Acupuncture and Tuina School, The Third Teaching Hospital, Chengdu University of Traditional Chinese Medicine, Chengdu, China; ^2^Acupuncture and Brain Science Research Center, Chengdu University of Traditional Chinese Medicine, Chengdu, China

**Keywords:** functional constipation, diffusion tensor imaging, white matter network, graph theory approach, visceral sensory process

## Abstract

**Background:** The abnormalities in brain function and structure of patients with functional constipation (FC) have been identified using multiple neuroimaging studies and have confirmed the abnormal processing of visceral sensation at the level of the central nervous system (CNS) as an important reason for FC. As an important basis for central information transfer, the role of the white matter (WM) networks in the pathophysiology of FC has not been investigated. This study aimed to explore the topological organization of WM networks in patients with FC and its correlation with clinical variables.

**Methods and Analysis:** In this study, 70 patients with FC and 45 age- and gender-matched healthy subjects (HS) were recruited. Diffusion tensor imaging (DTI) data and clinical variables were acquired from each participant. WM networks were constructed using the deterministic fiber tracking approach, and the global and nodal properties of the WM networks were compared using graph theory analysis between patients with FC and HS. The relationship between the representative nodal characteristics–nodal betweenness and clinical parameters was assessed using partial correlation analysis.

**Results:** Patients with FC showed increased nodal characteristics in the left superior frontal gyrus (orbital part), right middle frontal gyrus (orbital part), and right anterior cingulate and paracingulate (*P* < 0.05, corrected for false discovery rate) and decreased nodal characteristics in the left caudate and left thalamus (*P* < 0.05, corrected for false discovery rate) compared with HS. The duration of FC was negatively correlated with the nodal betweenness of the left thalamus (*r* = −0.354, *P* = 0.04, corrected for false discovery rate).

**Conclusion:** The results indicated the alternations in WM networks of patients with FC and suggested the abnormal visceral sensation processing in the CNS from the perspective of large-scale brain WM network.

## Introduction

Functional constipation (FC) is a common functional gastrointestinal disorder (FGID) with a global prevalence ranging approximately from 2 to 35% (1–3). FC is characterized by the following symptoms: straining, lumpy or hard stools, incomplete evacuation, and the sensation of anorectal obstruction (4, 5). Constipation impacts patients' quality of life (QoL) (6, 7) and increases healthcare costs on society and individuals (8, 9). Reports showed that nearly 5.7 million people were diagnosed with FC, which had led to a total annual cost of ~$235 million in the United States (10). Therefore, deeply investigating the underlying pathophysiology and developing effective treatment for FC are worthwhile.

The pathophysiology of FC is multifactorial and remains unclear, although altered motility (11, 12), visceral hypersensitivity (13), dysfunction of the brain–gut interaction (14), and psychosocial and behavioral factors (15, 16) have been proposed to explain the symptoms. Because the latest Rome IV criteria defined the FGIDs including FC as disorders of the brain–gut interaction (14), exploring the role of the central nervous system (CNS) in the pathophysiology of FGIDs by neuroimaging has received increasing attention.

Previous studies have found that patients with FC show abnormalities in both the brain function and structure compared with healthy subjects (HS). Multiple functional magnetic resonance imaging (fMRI) studies with different data analysis methods showed that patients with FC manifested significant brain activity alterations in the anterior insula and orbitofrontal cortex (OFC) (17), a stronger effective connectivity from the OFC and dorsal anterior cingulate cortex and a weaker effective connectivity from the supplementary motor area and precentral gyrus (18), and abnormal functional connectivity in the thalamocortical network. These results demonstrated that the functional abnormalities of patients with FC were not only in the brain regions but also in the functional networks (19). Additionally, using structural magnetic resonance imaging (sMRI), altered white matter (WM) microstructure (20) and cortical morphometry (21) in patients with FC have been identified. However, the features of structural connectivity especially WM network properties in patients with FC have not been covered.

Therefore, using diffusion tensor imaging (DTI) and graph theory method, this study aimed to (1) compare the topological characteristics of WM networks at the global and nodal levels between patients with FC and HS and (2) investigate the correlations between the topological characteristics of WM networks and clinical variables. The results might deepen our knowledge of the neural pathological mechanisms of FC.

## Materials and Methods

### Participants

#### Patients With FC

In this study, 70 patients with FC were recruited from the Digestive Department of the First Teaching Hospital of Chengdu University of Traditional Chinese Medicine (CDUTCM) and the campus of CDUTCM from September 2018 to January 2020. The patients were included if they (1) matched the Rome IV diagnostic criteria for FC, (2) were aged 20–40 years and were right handed, (3) had not taken a gastrointestinal prokinetic agent or laxative at least 15 days before enrollment, and (4) had not participated in other clinical trials in the past 3 months. The patients were excluded if they (1) suffered from cardiovascular, digestive, urinary, or hematopoietic system diseases and other organic diseases; (2) had a history of gastrointestinal surgery or head trauma with loss of consciousness; (3) had a history of mental disorders including major depressive disorders or anxiety disorders; (4) had a history of dysmenorrhea, being pregnant, lactating, or intending to get pregnant in the next 6 months; (5) had contraindication for MRI scanning; or (g) refused to sign an informed consent form.

#### Healthy Subjects

A total of 45 age- and gender-matched HS were recruited from the campus of CDUTCM from September 2018 to January 2020. The participants were included if they (1) were aged 20–40 years and were right handed, (2) were free from any organic or functional diseases via physical examination and laboratory test, and (3) had not participated in other clinical trials in the past 3 months. The participants were excluded if they (1) had any discomfort of the digestive system; (2) had a history of dysmenorrhea, being pregnant, lactating, or intending to get pregnant in the next 6 months; (3) had any gastrointestinal surgery or a history of head trauma with loss of consciousness; (4) had contraindication for MRI scanning; or (5) refused to sign an informed consent form.

This trial was approved by the Institutional Review Boards and Ethics Committees of the Affiliated Hospital of CDUTCM (Approved number: 2018KL-022, protocol version: F2.0) and was registered in the Chinese Clinical Trial Registry (http://www.chictr.org.cn/edit.aspx?pid=27816&htm=4) on August 9, 2018 (Registered number: ChiCTR1800017689).

### Clinical Evaluation

Symptom severity was assessed using the Cleveland Constipation Score (CCS) (22) and the Patient Assessment of Constipation Quality of Life Questionnaire (PAC-QoL) (23). Because variations of the patient's psychological status potentially lead to variations in the brain function and structure (24), this study used the Zung Self-Rating Anxiety Scale (SAS) (25), and the Zung Self-Rating Depression Scale (SDS) (26) to evaluate anxiety/depression-related symptoms of the participants.

### MRI Data Acquisition

All of the participants underwent MRI scans on a 3.0-T MRI scanner with an eight-channel phase-array head coil (GE 3.0 T MR750; GE Healthcare, Chicago, IL, USA) at the MRI Research Center of University of Electronic Science and Technology of China, Chengdu, China. During the MRI scans, all participants were instructed to keep their eyes closed and their head still. Earplugs and foam pads were used to decrease the noise interference and movement of the head, respectively. A high-resolution three-dimensional T1-weighted imaging (3D-T1WI), DTI was acquired with the following parameters: (1) the parameters of 3DT1 sequence using an axial fast spoiled gradient-recalled sequence were as follows: repetition time (TR)/echo time (TE) = 6.008 ms/1.98 ms, slice thickness = 1 mm, matrix size = 256 × 256, and field of view = 256 × 256 mm^2^ (2). The DTI sequence using a spin-echo imaging sequence included the following parameters: field of view = 256 × 256 mm^2^, TR/TE = 8,500 ms/minimum, matrix size = 128 × 128, slice thickness = 2 mm, and 78 slices with no gap. Two diffusion-weighted sequences were acquired using gradient values *b* = 0 s/mm^2^ and *b* = 1,000 s/mm^2^ with the diffusion-sensitizing gradients applied along 64 non-linear directions.

### MRI Data Preprocessing and Network Construction

#### MRI Data Preprocessing

Data preprocessing and brain WM network construction were conducted using PANDA software (http://www.nitrc.org/projects/panda/; a pipeline tool for diffusion MRI analysis) installed on the Linux system and MATLAB. The specific preprocessing steps were as follows: converted the imaging data to NIFTI format; skull removal with *BET*, correcting eddy current/motion, and calculating fractional anisotropy (FA) with DTIFIT; deterministic fiber tractography proceeded on the basis of the Fiber Assignment by Continuous Tracking (FACT) algorithm, and the propagation was terminated if an angle > 45° or the FA < 0.2 was encountered (27, 28); and registration to the Montreal Neurological Institute (MNI) space with a voxel size of 2 × 2 × 2 mm^3^.

#### Network Construction

The node is the most basic element of a network (29), which represents the brain regions with coherent patterns of extrinsic anatomical connections (30). To define the node of the networks, the Automated Anatomical Labeling (AAL) atlas was used to divide the whole brain into 90 cortical and subcortical regions (31). Each individual 3DT1 was registered to b0 image in the DTI space using a linear transformation and was then mapped into the MNI space using a non-linear transformation. The inverse matrix of the derived transformed result was used to warp the AAL atlas from the MNI space to the DTI space. The parcellation procedure has been applied previously (32–34).

The edge is another key element of the network; the mean FA of the connected streamlines between the two brain regions was defined as the weights of the edge (35). After that, a weighted and undirected symmetrical anatomical 90 × 90 matrix for each subject was obtained. The 90 brain regions are reported in Supplementary Material 1.

### Statistical Analysis

To investigate the topological properties of the WM networks at both the global and nodal levels, the GRETNA toolbox (http://www.nitrc.org/projects/gretna/) was applied (36).

#### Global Level Properties

This study included two kinds of global level properties: Small-world parameters contained clustering coefficient (C_p_) (37), characteristic path length (L_p_) (37), normalized clustering coefficient (γ) (38), normalized characteristic path length (λ) (38), and small worldness (σ) (37). A small-world network was defined with small worldness σ = γ/λ > 1, which fulfilled the conditions of γ (γ = C_p_/C_random_) > 1 and λ (λ = L_p_/L_random_) ≈ 1 (39) [C_random_ and L_random_ are the mean C_p_ and L_p_ of 100 matched random networks that preserve the same number of nodes, edges, and degree distribution as the real network (40)]. Network efficiency parameters contained local efficiency (E_loc_) (41) and global efficiency (E_glob_) (42). Integration and segregation represent two crucial information processing patterns of the complex network (30). Integration in the network means the efficiency of global information communication or the ability to integrate distributed information, which can be measured by the parameters L_p_, λ, and E_glob._ Conversely, the parameters C_p_, γ, and E_loc_ were used to reflect the segregation that referred to the specialized processing procedures in the network with a densely interconnected group of brain regions.

#### Nodal Level Characteristics

The characteristics of the nodal level used to evaluate the topological properties of the local brain regions in the networks were the nodal degree, nodal efficiency, and nodal betweenness. Nodal degree computed the sum of weights of edges connected to the node, which was a simple measurement of connectivity of a node with the rest of the nodes in the network (43). Nodal efficiency measured the information propagation ability of a node with the rest of the nodes in the network (42). Nodal betweenness measured the fraction of all the shortest paths in the network that passed through the node, which captured the influence of a node over information flow between other nodes in the network (30).

To construct a graph network, each connectivity matrix was converted into an undirected binary network using a connection sparsity (S), which is the ratio of the number of existing edges to all possible edges. Rather than setting a static sparsity value, thresholding each connectivity matrix can be performed repeatedly to increase the significance of the interpretation of topological property comparisons. Therefore, the criteria of the previous study were referenced (33), and then the range of S (0.1–0.34) was defined with an interval step of 0.01 for the WM connectivity network. For each network, the area under the curve (AUC) was calculated over the range of S values with an interval step of 0.01 and was compared across the two groups. The AUC provided a summarized scalar for the topological characterization of the brain network to limit potential bias of any single threshold. The AUC was sensitive to detect topological alterations in brain disorders (44, 45).

#### Clinical Data Analysis

For clinical variables, the two tailed independent-sample *t*-tests and chi-squared tests were used to compare continuous variables (age, duration, CCS, PAC-QoL, SAS, and SDS) and categorical variables (gender), respectively. Statistical significance was specified at 0.05, with all the *P*-values being two tailed. All the statistical analyses were performed using the SPSS software (USA, version 22.0).

#### WM Network Analysis

To examine the between-group differences in network metrics, non-parametric permutation tests were used to identify the AUCs of all the network metrics (small-world properties and network efficiency). The permutation test was repeated 10,000 times. The nodal characteristics were performed on the GRETNA based on MATLAB, and the false discovery rate (FDR) procedure was applied as multiple comparison corrections for nodal metric analysis (46). Age, gender, SAS, and SDS were used as covariates. Additionally, the node with the statistical difference commonly presented in nodal betweenness, nodal degree, and nodal efficiency would be found.

#### Correlation Analysis

A partial correlation analysis between neuroimaging index (nodal betweenness) and disease severity (duration and CCS) was performed, with age, gender, SAS, and SDS as covariates. FDR correction was applied for multiple comparisons. The correlation was performed on the brain regions with the statistical difference that were commonly presented in nodal betweenness, nodal degree, and nodal efficiency. Of the three nodal properties, nodal betweenness is more sensitive and effective to reflect the important control of information flow in the networks (30) and was chosen as the representative index to examine the correlation between the network metrics and the clinical variables (duration and CCS).

## Results

### Demographic and Clinical Data

Demographic characteristics (age and gender), clinical variables (duration, CCS, and PAC-QoL), and psychiatric measures (SAS and SDS) for two groups are reported in Table 1. No significant difference was found in age and gender between the two groups (*P* > 0.05). The SDS and SAS scores of patients with FC were significantly higher than HS (*P* < 0.05).

**Table 1:** Demographic and clinical characteristics of participants.

	**FC (*n =* 70) Mean ± SD**	**HS (*n =* 45) Mean ± SD**	***P*-value**
Age (years)	20.66 ± 1.99	21.38 ± 1.86	0.51^a^
Gender (male/female)	8/62	10/35	0.12^b^
Duration (months)	61.04 ± 29.44	/	
SAS	40.23 ± 8.24 Normal/Mild (52/18)	33.75 ± 5.95	0.00^*^^a^
SDS	43.91 ± 9.90 Normal/Mild (53/17)	35.17 ± 7.84	0.00^*^^a^
CCS	12.31 ± 2.00	/	
PAC-QOL	36.06 ± 12.67	/	

*FC, Functional Constipation group; HS, Healthy Subjects group; SAS, Self-rating Anxiety Scale; SDS, Self-rating Depression scale; CCS, Cleveland Constipation Score; PAC-QOL, Patient Assessment of Constipation Quality of Life Questionnaire*.

* *The marker indicated a significant difference in FC group and HS group*.

a *The P-value was obtained by two-tailed independent-sample t-tests*.

b *The P-value was obtained by chi-square test*.

### Global Topological Characteristics of WM Networks

**Figure 1:**
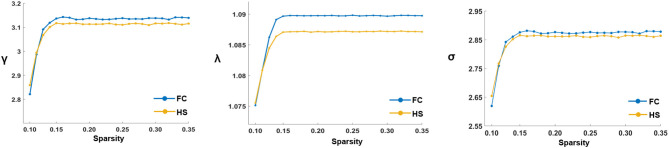
The key small-world parameters of the white matter networks in the defined sparsity threshold. Graphs showed that in the defined threshold range, both FC and HS groups exhibited γ obviously γ >> 1, λ ≈ 1 and σ = γ/λ > 1, which indicated that both groups exhibited the features of small-world topology. γ, Normalized clustering coefficient; λ, Normalized characteristic path length; σ, Small-worldness.

**Figure 2:**
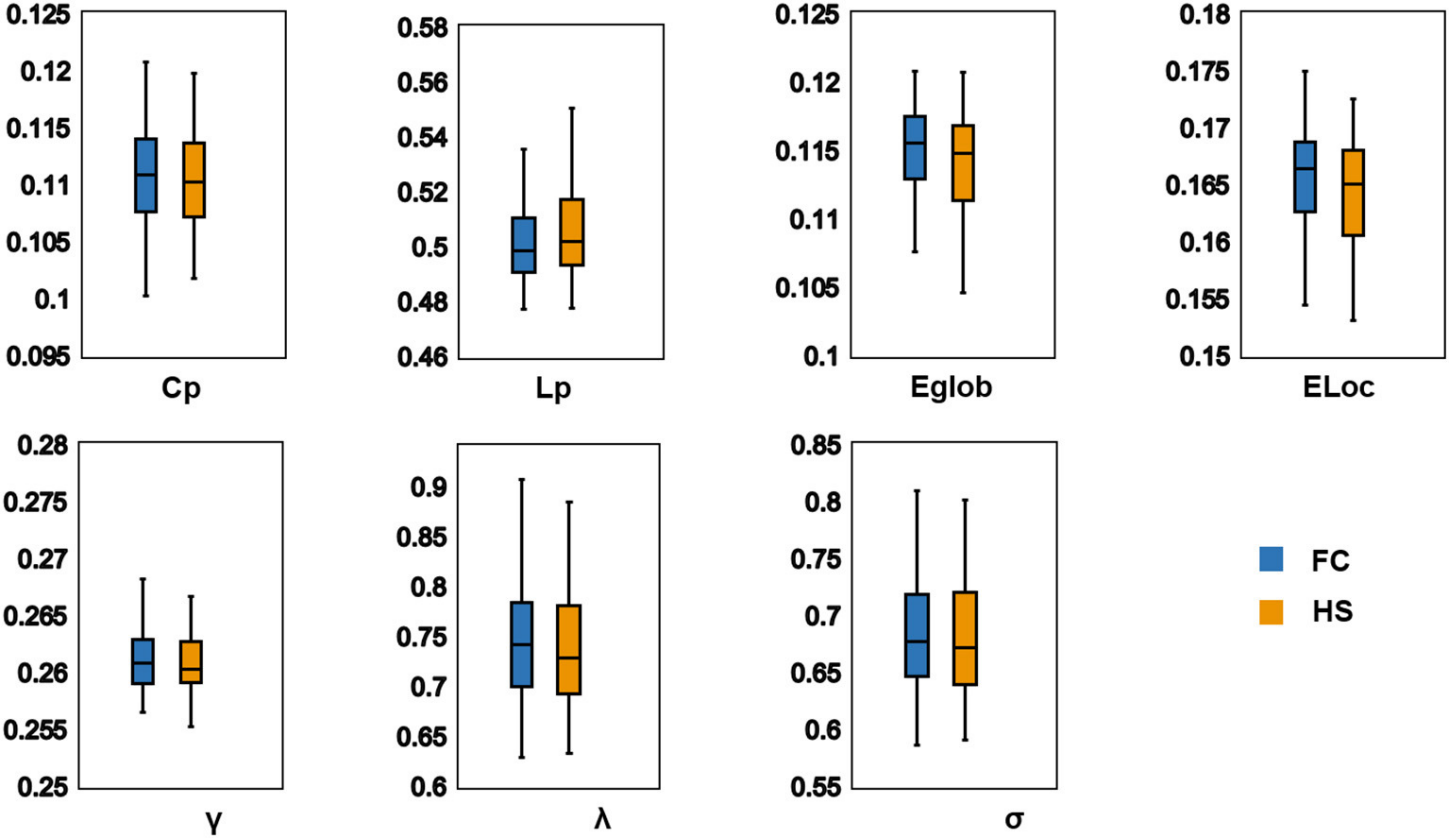
Differences in global topological characteristics of white matter networks between FC group and HS group. There were no significant difference between two groups. C_p_ (*P* = 0.396); L_p_ (*P* = 0.771); E_glob_ (*P* = 0.468); E_loc_ (*P* = 0.348); λ (*P* = 0.483); γ (*P* = 0.396); σ (*P* = 0.634). FC, Functional constipation; HC, Health control; C_p_, Clustering coefficient; γ, Normalized clustering coefficient; L_p_, Characteristic path length; λ, Normalized characteristic path length; E_glob_, Global efficiency; E_loc_, Local efficiency.

In the defined threshold range, both groups showed small-world network architecture in the WM networks (γ >> 1, λ ≈ 1, and σ = γ/λ > 1) (Figure 1). However, no significant differences were found between the two groups in these global topological characteristics including C_p_, L_p_, E_glob_, E_loc_, λ, γ, and σ (Figure 2).

### Nodal Topologic Characteristics of WM Networks

Compared with HS, patients with FC showed increased nodal betweenness in the left superior frontal gyrus (orbital part) (ORBsup.L), right middle frontal gyrus (orbital part) (ORBmid.R), and right anterior cingulate and paracingulate gyri (ACG.R); increased nodal efficiency in the ORBsup.L, right superior frontal gyrus (orbital part) (ORBsup.R), ORBmid.R, left anterior cingulate and paracingulate gyri (ACG.L), ACG.R, right amygdala (AMY.R), right lenticular nucleus (pallidum) (PAL.R), left superior temporal gyrus (temporal pole) (TPOsup.L), and the right superior temporal gyrus (temporal pole) (TPOsup.R); and increased nodal degree in the ORBsup.L, ORBmid.R, ACG.L, ACG.R, parahippocampal gyrus (PHG.R), lingual gyrus (LING.R), PAL.R, and TPOsup.L.

**Figure 3:**
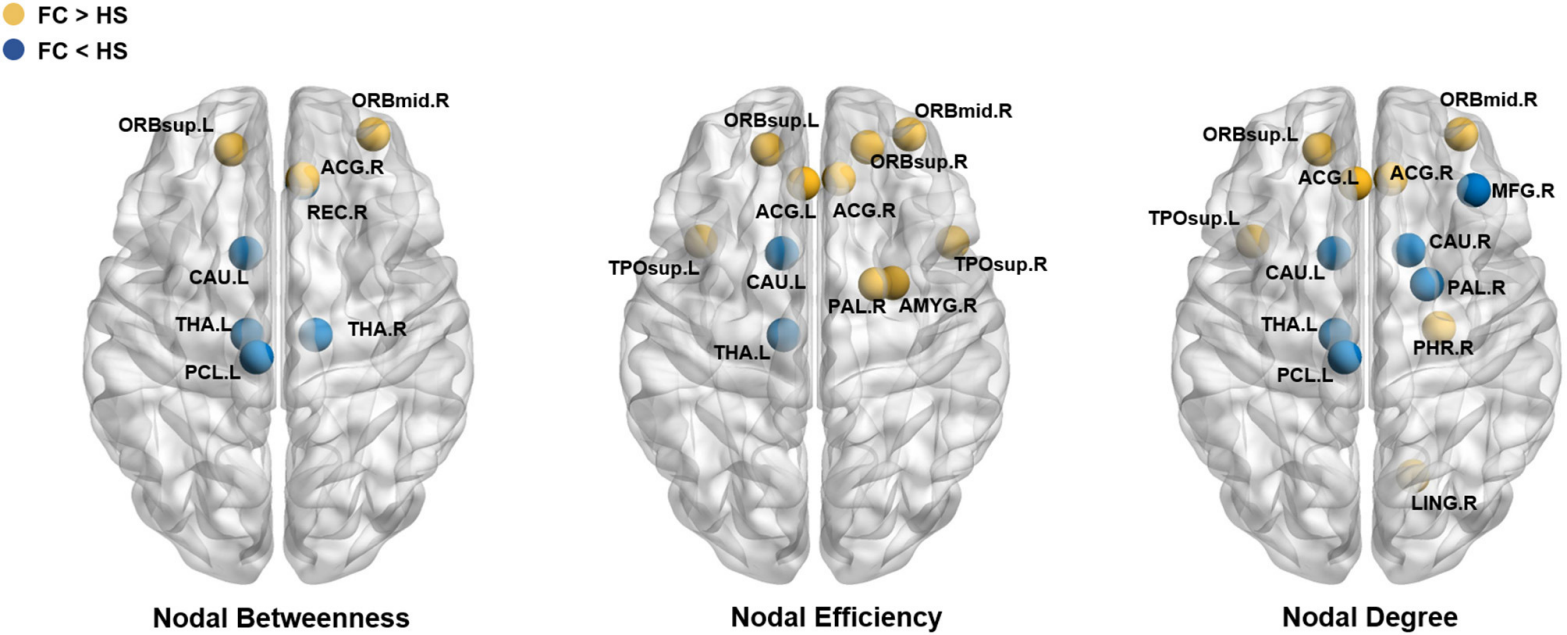
Brain regions showed significant difference in nodal characteristics between two groups (*P* < 0.05, FDR corrected). Every node denotes a brain region, the blue nodes represent the value of FC< the value of HS, the yellow nodes represent the value of FC > the value of HS. The abbreviations of the 90 brain regions were given in Supplementary Materials.

Patients with FC also showed decreased nodal betweenness in the right gyrus rectus (REC.R), left paracentral lobule (PCL.L), and left caudate (CAU.L); decreased nodal efficiency in the CAU.L and left thalamus (THA.L); and decreased nodal degree in the right middle frontal gyrus (MFG.R), PCL.L, CAU.L, right caudate (CAU.R), and THA.L (Table 2 and Figure 3). The detailed results and node abbreviations correspond to those that are given in Supplementary Material 2.

**Table 2:** The significant differences of nodal characteristics existed in between group.

**Brain regions**	**Category**	**Nodal betweenness Mean ± SD**			**Nodal degree Mean ± SD**			**Nodal efficiency Mean ± SD**		
		**FC**	**HS**	***P***	**FC**	**HS**	***P***	**FC**	**HS**	***P***
**FC>HS**										
**Frontal_Sup_Orb_L**	**Paralimbic**	**27.83 ± 16.53**	**16.40 ± 14.07**	0.0002^*^	**3.64 ± 0.80**	**2.83 ± 1.03**	0.0003^*^	**0.124 ± 0.008**	**0.117 ± 0.012**	0.0001^*^
Frontal_Sup_Orb_R	Paralimbic							0.123 ± 0.007	0.116 ± 0.013	0.0003^*^
**Frontal_Mid_Orb_R**	**Paralimbic**	**11.72 ± 8.99**	**5.64 ± 6.20**	0.0036^*^	**2.64 ± 0.72**	**1.86 ± 0.95**	0.0001^*^	**0.117 ± 0.009**	**0.108 ± 0.012**	0.0000^*^
Cingulum_Ant_L	Paralimbic				2.74 ± 0.46	2.13 ± 0.63	0.0000^*^	0.117 ± 0.006	0.113 ± 0.009	0.0008^*^
**Cingulum_Ant_R**	**Paralimbic**	**14.12 ± 6.21**	**7.88 ± 5.42**	0.0000^*^	**2.74 ± 0.46**	**2.05 ± 0.62**	0.0000^*^	**0.119 ± 0.006**	**0.113 ± 0.008**	0.0000^*^
ParaHippocampal_R	Paralimbic				2.24 ± 0.51	1.97 ± 0.42	0.0067^*^			
Amygdala_R	Subcortical							0.098 ± 0.007	0.093 ± 0.010	0.0005^*^
Lingual_R					2.82 ± 0.63	2.54 ± 0.60	0.0071			
Pallidum_R	Subcortical				1.55 ± 0.59	1.16 ± 0.63	0.0014^*^	0.108 ± 0.009	0.102 ± 0.010	0.0013^*^
Temporal_Pole_Sup_L	Association				3.46 ± 0.81	2.92 ± 0.82	0.0026^*^	0.121 ± 0.008	0.115 ± 0.009	0.0003^*^
Temporal_Pole_Sup_R	Association							0.119 ± 0.007	0.113 ± 0.010	0.0002^*^
**FC < HS**										
Frontal_Mid_R	Association				1.80 ± 0.52	2.23 ± 0.66	0.0004^*^			
Rectus_R	Paralimbic	5.56 ± 4.35	11.14 ± 8.18	0.0002^*^						
Paracentral_Lobule_L	Association	7.33 ± 3.98	12.42 ± 10.01	0.0022	2.23 ± 0.53	2.65 ± 0.75	0.0029^*^			
**Caudate_L**	**Subcortical**	**13.18 ± 10.11**	**26.84 ± 16.64**	0.0000^*^	**2.62 ± 0.65**	**3.21 ± 0.79**	0.0020^*^	**0.118 ± 0.008**	**0.124 ± 0.008**	0.0003^*^
Caudate_R	Subcortical				2.53 ± 0.68	3.10 ± 0.73	0.0058^*^			
**Thalamus_L**	**Subcortical**	**25.27 ± 14.64**	**38.92 ± 17.53**	0.0000^*^	**3.30 ± 1.02**	**3.87 ± 0.68**	0.0018^*^	**0.126 ± 0.011**	**0.132 ± 0.007**	0.0011^*^
Thalamus_R	Subcortical	17.86 ± 11.15	27.85 ± 15.37	0.0030^*^						

*FC, Functional Constipation group; HS, Healthy Subjects group. The abbreviations of the 90 brain regions are given in Supplementary Materials*.

* *The marker indicated a significant difference in FC group and HS group. P < 0.05, FDR corrected*.

*The bold black font denotes that differences existed in the three nodal characteristics simultaneously*.

**Figure 4:**
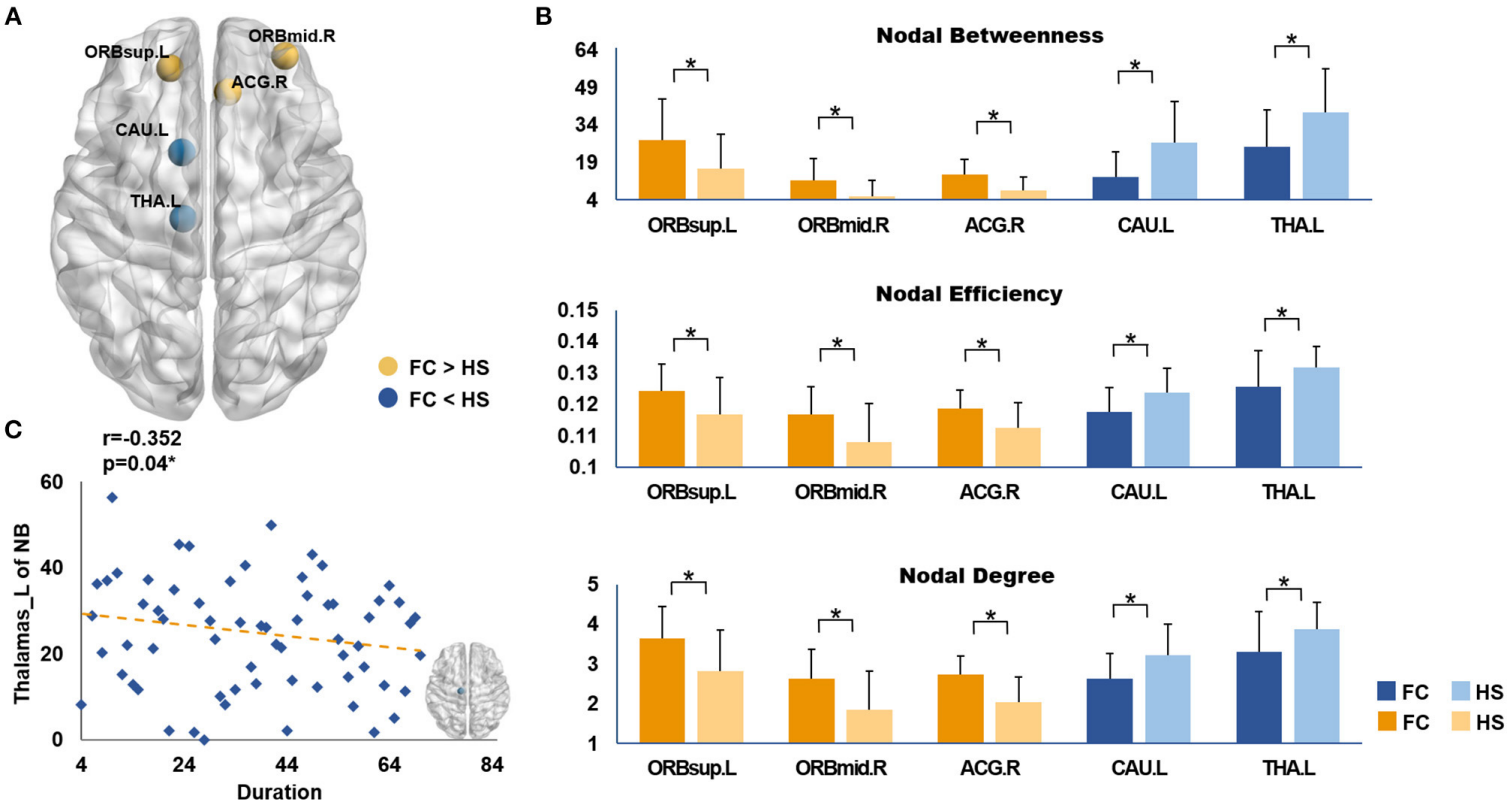
**(A)** Brain regions showed the overlap nodal characteristics of significant difference; **(B)** The differences in nodal topological properties between FC and HS. The marks (*) indicated statistically significant differences between the two groups (*P* < 0.05, FDR corrected). **(C)** The correlations between duration and the nodal betweenness of left thalamus in patients with FC. FC, Functional constipation; HS, Health Subjects; The abbreviations of the 90 brain regions were given in Supplementary Materials.

After analyzing the results of the nodal topologic characteristics, the overlap regions were found. Patients with FC exhibited increased nodal characteristics in the ORBsup.L, ORBmid.R, and ACG.R, whereas decreased nodal characteristics were found in the CAU.L and THA.L compared with HS (*P* < 0.05, FDR corrected; Figures 4A,B).

### Correlations Between WM Networks and Clinical Variables

Correlation analysis showed that disease duration (months) was negatively correlated with the nodal betweenness in the THA.L (*r* = −0.352, *P* = 0.04, FDR corrected; Figure 4C). No significant correlation was found between the CCS and the nodal characteristics of the overlapped nodes.

## Discussion

This study is the first study exploring the topological organization of WM networks in patients with FC. The results indicated the significant differences at the nodal level in the WM networks between patients with FC and HS, providing evidence for cerebral structural alterations.

WM comprises half of the brain (47) and serves as important material foundations for information transfer across brain regions (48). The topological characteristics of WM networks not only represent how the brain is structurally organized but also provide the structural topology involving the information processing ability of the brain (49). In the global level analysis of this study, both patients with FC and HS manifested small-world network architecture (σ = γ/λ > 1, with γ > 1 and λ ≈ 1) in the WM networks. The small-world network architecture is one of the consistent topological characteristics of the structural brain networks (49). It has both high local and global efficiencies, which meant an optimal balance between the segregation and integration of information processing procedures (38, 50, 51). A study also showed that the functional networks in patients with FC manifested small-world network architecture (19). These results suggested that patients with FC exhibited small-world network architecture in both cerebral functional and structural networks. However, this study did not find significant group differences in small-world indexes (C_p_, L_p_, γ, λ, and σ) and network efficiency (E_glob_ and E_loc_) in the WM networks, although some study found that the γ and σ values of patients with FC were significantly lower than those of HS in the functional networks (19). The current results indicated that no altered capacities of information transformation were observed in the global level of the WM networks. Possibly, this may result from the mutual compensation of information flows in the global level of the WM networks to maximize the efficiency of information processed in patients with FC.

In the nodal level analysis of this study, three commonly used indexes including nodal degree, nodal efficiency, and nodal betweenness were selected to describe the nodal characteristics. Regional nodal characteristics are used to identify network hubs that are critical to establish and maintain efficient information transfer across regions (52). The significantly different results of the three nodal characteristics that described the measurement of connectivity (nodal degree), information propagation ability (nodal efficiency), and information flow (nodal betweenness) of the nodes were presented simultaneously; the overlapped nodes were summarized in this study. This study found that patients with FC have increased nodal characteristics in the ORBsup.L, ORBmid.R, and ACG.R and decreased nodal characteristics in the CAU.L and THA.L compared with HS.

### The Increased Nodal Characteristics in Patients With FC

In the study, the increased nodal characteristics of patients with FC were mainly manifested in the paralimbic areas including the ORBsup.L, ORBmid.R, and ACG.R. The ORBsup.L and ORBmid.R are parts of the OFC. The ACG receives the projection from the OFC (53). The OFC and ACG are thought to be neural bridges that link the external environment to the internal milieu integrating the process of visceral sensation information (54, 55).

As a key region involved in the conduction and integration of sensory information, OFC receives multiple sensory inputs (gustatory, olfactory, somatosensory, auditory, and visual) and visceral sensory information (56) and is considered to be important in integrating visceral sensorimotor information (57, 58). Functional and structural abnormalities of the OFC in patients with FC were found in some neuroimaging studies (17, 21). For example, a recent structural MRI study showed that patients with FC manifested abnormal cortical thickness of the OFC compared with HS (21), and the fMRI study found altered activity of the same structure (18).

The ACG plays an important role in the process of gastrointestinal sensory signals (59) and visceromotor control (60). Similar to OFC, functional and structural alterations of the ACG were found in FC neuroimaging studies. For example, patients with FC showed significantly increased activity in the ACG with the amplitude of low-frequency fluctuation analysis (18) and altered nodal degree and efficiency in the ACG with functional network analysis compared with HS (19). Furthermore, the stronger functional effective connectivity of the OFC and ACG was detected among patients with FC, which is related to the abnormal regulating visceral response (18). Examining the current results, these findings suggested a significant contribution of the OFC and ACG to the central pathogenies of FC.

The OFC and ACG belong to the paralimbic areas that mediate the affective and cognitive components of the visceral sensation (55). In this study, the increasing nodal characteristics of the OFC and ACG were found in the WM networks. The expectation of defecation resulting in abnormal visceral sensory integration was speculated to cause the compensational increasing connectivity, information propagation ability, and flow in the paralimbic areas. The results provided new evidence of the abnormal process in visceral modulation and sensory transduction in the paralimbic areas particularly the OFC and ACG in FC.

### The Decreased Nodal Characteristics in Patients With FC

In this study, decreased nodal characteristics in the CAU.L and THA.L of patients with FC were found compared with those of HS. The caudate is regarded as a component of the subcortical apparatus of the integrative function in the brain (61) and plays a role in the interaction of the visceral sensory with the somatic and auditory systems (62, 63). As a central relay region, the thalamus relays visceral inputs to the cortical components of the “visceral sensory neuromatrix” (64–66) and participates in the gastrointestinal sensory modulation. The functional and structural alterations in the caudate and thalamus were found in multiple FGIDs including irritable bowel syndrome (67, 68), functional dyspepsia (69, 70), and FC (19). In this study, the decreased nodal characteristics in the caudate and thalamus indicated the disrupted and less interregional information flows of the visceral sensory, which would have an impact on the processing of the visceral sensory in patients with FC. The results emphasized the important role of the thalamus in the structural network of FC.

Furthermore, the correlation analysis showed a negative association between the nodal betweenness in the thalamus and the duration of symptoms in patients with FC (FDR corrected), indicating that longer FC duration is related to lower information flows between THA.L and other nodes in WM networks. The result suggested that the decreased information flows of THA.L may be involved in the illness progression of FC. It supported the notion of neuronal plasticity that referred to structural and functional changes in neuronal circuits in response to experience (71); that is, the experience of long disease duration may be associated with more profound structural changes, and the alterations of structural changes (WM networks) might account for brain functional changes in patients with FC. However, the CCS is not associated with the nodal characteristics, which may account for the mild symptom of patients with averaged scores of 12 (22).

In conclusion, this study demonstrated the significantly different nodal characteristics in the OFC, ACG, caudate, and thalamus in the WM networks in patients with FC compared with those in HS using DTI combined with the graph theory method. The results revealed the alterations in the WM networks of patients with FC and suggested abnormal visceral sensation processing in the CNS. Although the causal link between the cerebral structural abnormalities in WM networks and symptom occurrence remains unclear and is worth investigating, the results further provided the visualized evidence of FC accompanied by cerebral structural alterations and deepen our knowledge on the pathology of FGIDs.

## Data Availability Statement

The raw data supporting the conclusions of this article will be made available by the authors, without undue reservation.

## Ethics Statement

The studies involving human participants were reviewed and approved by the Institutional Review Boards and Ethics Committees of the Affiliated Hospital of CDUTCM (Approved number: 2018KL-022, protocol version: F2.0) and had been registered in Chinese Clinical Trial Registry (http://www.chictr.org.cn/edit.aspx?pid=27816&amp;htm=4) on Aug 9, 2018 (Registered number: ChiCTR1800017689). The patients/participants provided their written informed consent to participate in this study.

## Author Contributions

ZF was responsible for this study. ZF, SR, and HZ designed the study. YS, XK, GY, and WF were in charge of recruiting participants. CJ and YT contributed to the acquisition of imaging data. CL and HL were responsible for the data collation. MP, YT, and TZ were responsible for the data analysis. YT, MP, and HZ drafted the manuscript. SR and ZF revised the manuscript. All authors approved the final manuscript.

## Conflict of Interest

The authors declare that the research was conducted in the absence of any commercial or financial relationships that could be construed as a potential conflict of interest.

## Supplementary Material

Supplementary Figure 1 **(A)** Brain regions showed the nodal betweenness of significant difference; **(B,C)** The differences in nodal betweenness between FC and HS. The marks (^*^) indicated statistically significant differences between the two groups (*P* < 0.05, FDR corrected).

Supplementary Figure 2 **(A)** Brain regions showed the nodal efficiency of significant difference; **(B,C)** The differences in nodal efficiency between FC and HS. The marks (^*^) indicated statistically significant differences between the two groups (*P* < 0.05, FDR corrected).

Supplementary Figure 3 **(A)** Brain regions showed the nodal degree of significant difference; **(B,C)** The differences in nodal degree between FC and HS. The marks (^*^) indicated statistically significant differences between the two groups (*P* < 0.05, FDR corrected).

## Abbreviations

FC: Functional Constipation
FGIDs: Functional gastrointestinal disorder
QoL: Quality of life
CNS: Central nervous system
HS: Healthy subjects
fMRI: functional Magnetic Resonance Imaging
OFC: Orbital frontal cortex
sMRI: structural magnetic resonance imaging
WM: White Matter
DTI: Diffusion Tensor Imaging
CDUTCM: Chengdu University of Traditional Chinese Medicine
CCS: Cleveland Constipation Score
PAC-QoL: Patient Assessment of Constipation Quality of Life Questionnaire
SAS: the Zung Self-Rating Anxiety Scale
SDS: the Zung Self-Rating Depression Scale
3D-T1WI: 3-Dimensional T1-Weighted Imaging
TR: Repetition time
TE: Echo time
FA: Fractional anisotropy
MNI: Montreal Neurological Institute
AAL: Automated Anatomical Labeling
C_p_: Clustering coefficient
L_p_: Characteristic path length
γ: Normalized clustering coefficient
λ: Normalized characteristic path length
σ: Small-worldness
E_loc_: Local efficiency
E_glob_: Global efficiency
AUC: Area under the curve
FDR: False discovery rate
ORBsup. L: left superior frontal gyrus (orbital part)
ORBmid.R: right middle frontal gyrus (orbital part)
ACG.R: right anterior cingulate and paracingulate gyri
ORBsup.R: right superior frontal gyrus (orbital part)
ACG.L: left anterior cingulate and paracingulate gyri
AMY.R: right amygdala
PAL.R: right lenticular nucleus (pallidum)
TPOsup.L: left superior temporal gyrus (temporal pole)
TPOsup.R: right superior temporal gyrus (temporal pole)
PHG.R: parahippocampal gyrus
LING.R: lingual gyrus
REC.R: right gyrus rectus
PCL.L: left paracentral lobule
CAU.L: left caudate
THA.L: left thalamus
MFG.R: right middle frontal gyrus
CAU.R: right caudate.
