# Supplementary material for: Alterations of White Matter Network Properties in Patients With Functional Constipation
Source: Front Neurol. 2021 Mar 24;12:627130. doi: 10.3389/fneur.2021.627130 (PMC8024587; doi:10.3389/fneur.2021.627130)
Supplement: Supplementary file 1 [file Table_1.DOCX]

**Supplementary 1**

| Labels | Full Name of Brain Regions | Brain Regions | Abbreviation |
| --- | --- | --- | --- |
| 1 | Precental gyrus | Precentral_L | PreCG.L |
| 2 | Precental gyrus | Precentral_R | PreCG.R |
| 3 | Superior frontal gyrus, dorsolateral | Frontal_Sup_L | SFGdor.L |
| 4 | Superior frontal gyrus, dorsolateral | Frontal_Sup_R | SFGdor.R |
| 5 | Superior frontal gyrus, orbital part | Frontal_Sup_Orb_L | ORBsup.L |
| 6 | Superior frontal gyrus, orbital part | Frontal_Sup_Orb_R | ORBsup.R |
| 7 | Middle frontal gyrus | Frontal_Mid_L | MFG.L |
| 8 | Middle frontal gyrus | Frontal_Mid_R | MFG.R |
| 9 | Middle frontal gyrus, orbital part | Frontal_Mid_Orb_L | ORBmid.L |
| 10 | Middle frontal gyrus, orbital part | Frontal_Mid_Orb_R | ORBmid.R |
| 11 | Inferior frontal gyrus, opercular part | Frontal_Inf_Oper_L | IFGoperc.L |
| 12 | Inferior frontal gyrus, opercular part | Frontal_Inf_Oper_R | IFGoperc.R |
| 13 | Inferior frontal gyrus, triangular part | Frontal_Inf_Tri_L | IFGtriang.L |
| 14 | Inferior frontal gyrus, triangular part | Frontal_Inf_Tri_R | IFGtriang.R |
| 15 | Inferior frontal gyrus, orbital part | Frontal_Inf_Orb_L | ORBinf.L |
| 16 | Inferior frontal gyrus, orbital part | Frontal_Inf_Orb_R | ORBinf.R |
| 17 | Rolandic operculum | Rolandic_Oper_L | ROL.L |
| 18 | Rolandic operculum | Rolandic_Oper_R | ROL.R |
| 19 | Supplementary motor area | Supp_Motor_Area_L | SMA.L |
| 20 | Supplementary motor area | Supp_Motor_Area_R | SMA.R |
| 21 | Olfactory cortex | Olfactory_L | OLF.L |
| 22 | Olfactory cortex | Olfactory_R | OLF.R |
| 23 | Superior frontal gyrus, medial | Frontal_Sup_Medial_L | SFGmed.L |
| 24 | Superior frontal gyrus, medial | Frontal_Sup_Medial_R | SFGmed.R |
| 25 | Superior frontal gyrus, medial orbital | Frontal_Mid_Orb_L | ORBsupmed.L |
| 26 | Superior frontal gyrus, medial orbital | Frontal_Mid_Orb_R | ORBsupmed.R |
| 27 | Gyrus rectus | Rectus_L | REC.L |
| 28 | Gyrus rectus | Rectus_R | REC.R |
| 29 | Insula | Insula_L | INS.L |
| 30 | Insula | Insula_R | INS.R |
| 31 | Anterior cingulate and paracingulate gyri | Cingulum_Ant_L | ACG.L |
| 32 | Anterior cingulate and paracingulate gyri | Cingulum_Ant_R | ACG.R |
| 33 | Median cingulate and paracingulate gyri | Cingulum_Mid_L | DCG.L |
| 34 | Median cingulate and paracingulate gyri | Cingulum_Mid_R | DCG.R |
| 35 | Posterior cingulate gyrus | Cingulum_Post_L | PCG.L |
| 36 | Posterior cingulate gyrus | Cingulum_Post_R | PCG.R |
| 37 | Hippocampus | Hippocampus_L | HIP.L |
| 38 | Hippocampus | Hippocampus_R | HIP.R |
| 39 | Parahippocampal gyrus | ParaHippocampal_L | PHG.L |
| 40 | Parahippocampal gyrus | ParaHippocampal_R | PHG.R |
| 41 | Amygdala | Amygdala_L | AMYG.L |
| 42 | Amygdala | Amygdala_R | AMYG.R |
| 43 | Calcarine fissure and surrounding cortex | Calcarine_L | CAL.L |
| 44 | Calcarine fissure and surrounding cortex | Calcarine_R | CAL.R |
| 45 | Cuneus | Cuneus_L | CUN.L |
| 46 | Cuneus | Cuneus_R | CUN.R |
| 47 | Lingual gyrus | Lingual_L | LING.L |
| 48 | Lingual gyrus | Lingual_R | LING.R |
| 49 | Superior occipital gyrus | Occipital_Sup_L | SOG.L |
| 50 | Superior occipital gyrus | Occipital_Sup_R | SOG.R |
| 51 | Middle occipital gyrus | Occipital_Mid_L | MOG.L |
| 52 | Middle occipital gyrus | Occipital_Mid_R | MOG.R |
| 53 | Inferior occipital gyrus | Occipital_Inf_L | IOG.L |
| 54 | Inferior occipital gyrus | Occipital_Inf_R | IOG.R |
| 55 | Fusiform gyrus | Fusiform_L | FFG.L |
| 56 | Fusiform gyrus | Fusiform_R | FFG.R |
| 57 | Postcentral gyrus | Postcentral_L | PoCG.L |
| 58 | Postcentral gyrus | Postcentral_R | PoCG.R |
| 59 | Superior parietal gyrus | Parietal_Sup_L | SPG.L |
| 60 | Superior parietal gyrus | Parietal_Sup_R | SPG.R |
| 61 | Inferior parietal, but supramarginal and angular gyri | Parietal_Inf_L | IPL.L |
| 62 | Inferior parietal, but supramarginal and angular gyri | Parietal_Inf_R | IPL.R |
| 63 | Supramarginal gyrus | SupraMarginal_L | SMG.L |
| 64 | Supramarginal gyrus | SupraMarginal_R | SMG.R |
| 65 | Angular gyrus | Angular_L | ANG.L |
| 66 | Angular gyrus | Angular_R | ANG.R |
| 67 | Precuneus | Precuneus_L | PCUN.L |
| 68 | Precuneus | Precuneus_R | PCUN.R |
| 69 | Paracentral lobule | Paracentral_Lobule_L | PCL.L |
| 70 | Paracentral lobule | Paracentral_Lobule_R | PCL.R |
| 71 | Caudate nucleus | Caudate_L | CAU.L |
| 72 | Caudate nucleus | Caudate_R | CAU.R |
| 73 | Lenticular nucleus, putamen | Putamen_L | PUT.L |
| 74 | Lenticular nucleus, putamen | Putamen_R | PUT.R |
| 75 | Lenticular nucleus, pallidum | Pallidum_L | PAL.L |
| 76 | Lenticular nucleus, pallidum | Pallidum_R | PAL.R |
| 77 | Thalamus | Thalamus_L | THA.L |
| 78 | Thalamus | Thalamus_R | THA.R |
| 79 | Heschl gyrus | Heschl_L | HES.L |
| 80 | Heschl gyrus | Heschl_R | HES.R |
| 81 | Superior temporal gyrus | Temporal_Sup_L | STG.L |
| 82 | Superior temporal gyrus | Temporal_Sup_R | STG.R |
| 83 | Temporal pole: superior temporal gyrus | Temporal_Pole_Sup_L | TPOsup.L |
| 84 | Temporal pole: superior temporal gyrus | Temporal_Pole_Sup_R | TPOsup.R |
| 85 | Middle temporal gyrus | Temporal_Mid_L | MTG.L |
| 86 | Middle temporal gyrus | Temporal_Mid_R | MTG.R |
| 87 | Temporal pole: middle temporal gyrus | Temporal_Pole_Mid_L | TPOmid.L |
| 88 | Temporal pole: middle temporal gyrus | Temporal_Pole_Mid_R | TPOmid.R |
| 89 | Inferior temporal gyrus | Temporal_Inf_L | ITG.L |
| 90 | Inferior temporal gyrus | Temporal_Inf_R | ITG.R |
