# Supplementary material for: Alterations of White Matter Network Properties in Patients With Functional Constipation
Source: Front Neurol. 2021 Mar 24;12:627130. doi: 10.3389/fneur.2021.627130 (PMC8024587; doi:10.3389/fneur.2021.627130)
Supplement: Supplementary Figure 1 — (A) Brain regions showed the nodal betweenness of significant difference; (B,C) The differences in nodal betweenness between FC and HS. The marks (*) indicated statistically significant differences between the two groups (P < 0.05, FDR corrected). [file Presentation_1.pptx]

## Slide 1
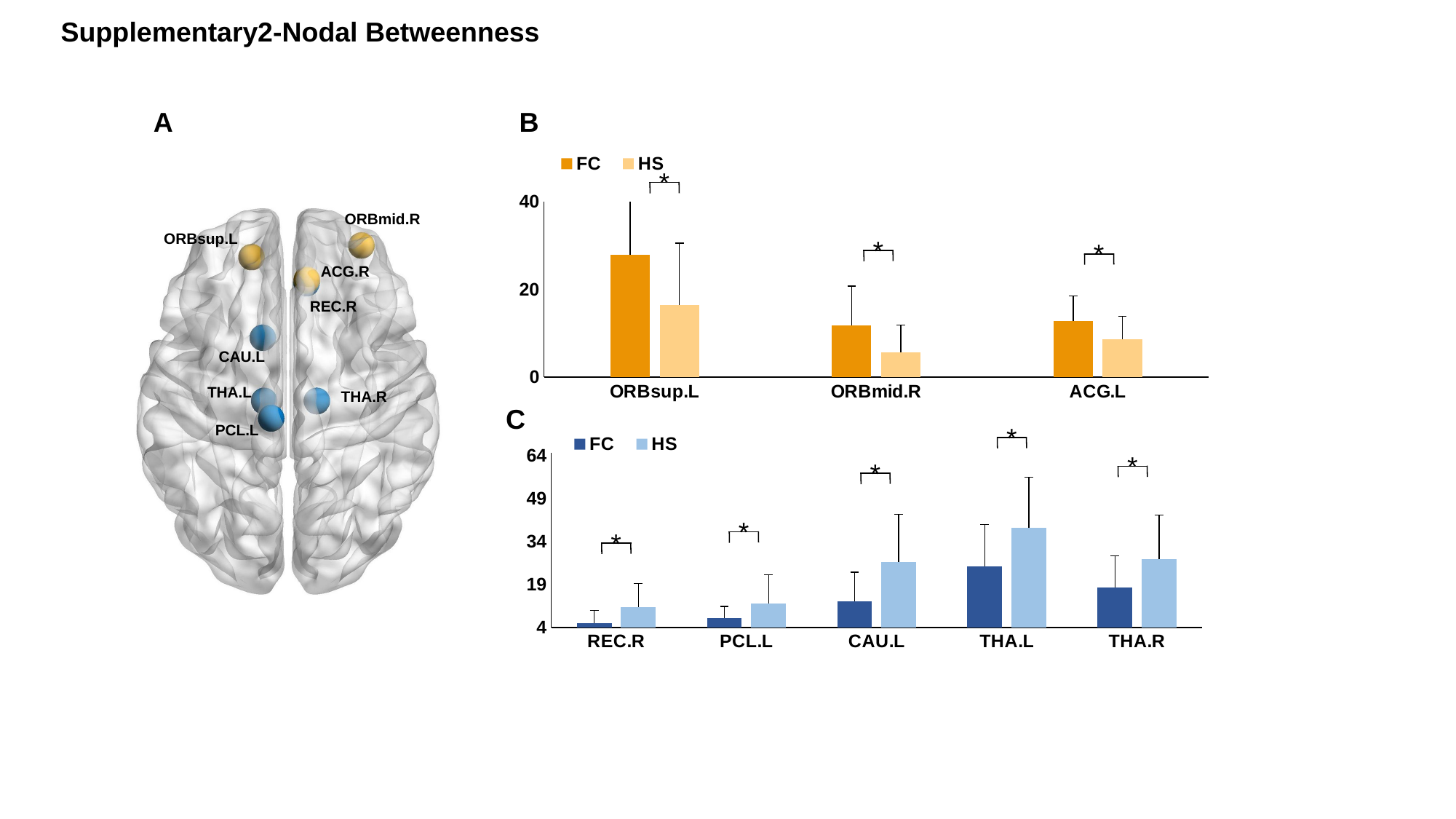

Supplementary2-Nodal Betweenness
A
B
### Chart
| Category | FC | HS |
|---|---|---|
| ORBsup.L | 27.83 | 16.4 |
| ORBmid.R | 11.72 | 5.64 |
| ACG.L | 12.81 | 8.69 |*
ORBmid.R
ORBsup.L
ACG.R
REC.R
CAU.L
THA.L
THA.R
PCL.L
*
*
C
*
### Chart
| Category | FC | HS |
|---|---|---|
| REC.R | 5.56 | 11.14 |
| PCL.L | 7.33 | 12.42 |
| CAU.L | 13.18 | 26.84 |
| THA.L | 25.27 | 38.92 |
| THA.R | 17.86 | 27.85 |*
*
*
*

## Slide 2
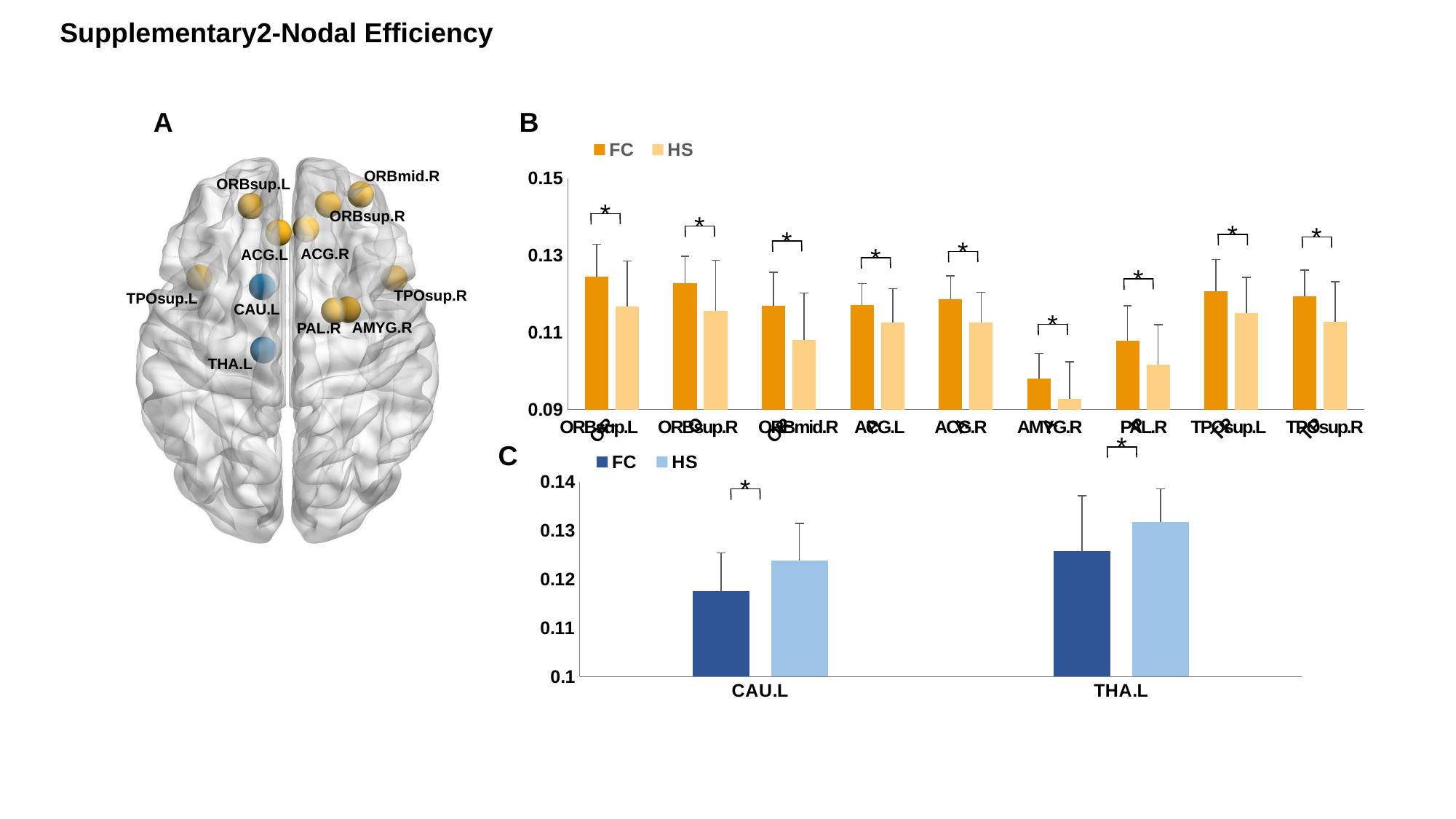

Supplementary2-Nodal Efficiency
A
B
ORBmid.R
ORBsup.L
ORBsup.R
ACG.R
ACG.L
TPOsup.L
CAU.L
AMYG.R
PAL.R
THA.L
TPOsup.R
### Chart
| Category | FC | HS |
|---|---|---|
| ORBsup.L | 0.1244 | 0.1168 |
| ORBsup.R | 0.1227 | 0.1156 |
| ORBmid.R | 0.1169 | 0.108 |
| ACG.L | 0.1171 | 0.1126 |
| ACG.R | 0.1186 | 0.1126 |
| AMYG.R | 0.098 | 0.0927 |
| PAL.R | 0.1078 | 0.1016 |
| TPOsup.L | 0.1207 | 0.1151 |
| TPOsup.R | 0.1193 | 0.1128 |ORBsup.L ORBsup.R ORBmid.R ACG.L ACG.R AMYG.R PAL.R TPOsup.L TPOsup.R
*
*
*
*
*
*
*
*
*
### Chart
| Category | FC | HS |
|---|---|---|
| CAU.L | 0.1175 | 0.1238 |
| THA.L | 0.1257 | 0.1317 |*
*
C

## Slide 3
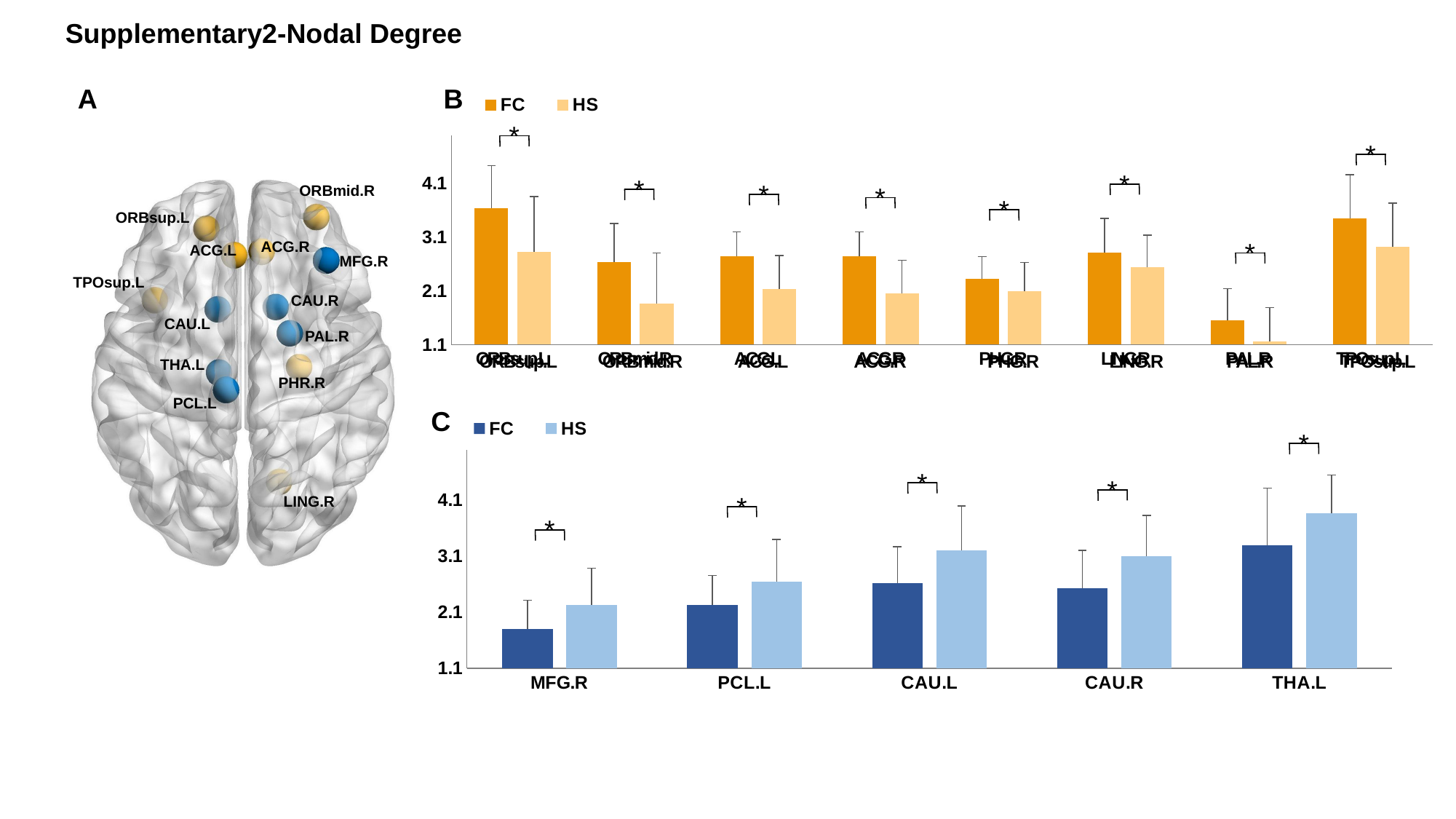

Supplementary2-Nodal Degree
A
B
### Chart
| Category | FC | HS |
|---|---|---|
| ORBsup.L | 3.64 | 2.83 |
| ORBmid.R | 2.64 | 1.86 |
| ACG.L | 2.74 | 2.13 |
| ACG.R | 2.74 | 2.05 |
| PHG.R | 2.32 | 2.09 |
| LING.R | 2.82 | 2.54 |
| PAL.R | 1.55 | 1.16 |
| TPOsup.L | 3.46 | 2.92 |ORBsup.L ORBmid.R ACG.L ACG.R PHG.R LING.R PAL.R TPOsup.L
*
*
*
*
*
*
*
*
ORBmid.R
ORBsup.L
ACG.R
ACG.L
TPOsup.L
CAU.L
PAL.R
THA.L
PHR.R
MFG.R
CAU.R
PCL.L
### Chart
| Category | FC | HS |
|---|---|---|
| MFG.R | 1.8 | 2.23 |
| PCL.L | 2.23 | 2.65 |
| CAU.L | 2.62 | 3.21 |
| CAU.R | 2.53 | 3.1 |
| THA.L | 3.3 | 3.87 |*
*
*
*
*
C
LING.R
